# Gravid *Anopheles gambiae* sensu stricto avoid ovipositing in Bermuda grass hay infusion and it’s volatiles in two choice egg-count bioassays

**DOI:** 10.1186/s12936-016-1330-6

**Published:** 2016-05-12

**Authors:** Lynda K. Eneh, Michael N. Okal, Anna-Karin Borg-Karlson, Ulrike Fillinger, Jenny M. Lindh

**Affiliations:** Chemical Ecology, Department of Chemistry, School of Chemical Science and Engineering, Royal Institute of Technology, Stockholm, Sweden; International Centre of Insect Physiology and Ecology, Thomas Odhiambo Campus, Mbita, Kenya; Disease Control Department, London School of Hygiene & Tropical Medicine, London, UK

**Keywords:** *Anopheles gambiae*, Oviposition behaviour, Egg-count cage bioassays, Hay infusions, Bermuda grass, Volatile detection, GC–MS

## Abstract

**Background:**

A number of mosquito species in the *Culex* and *Aedes* genera prefer to lay eggs in Bermuda grass (*Cynodon dactylon*) hay infusions compared to water alone. These mosquitoes are attracted to volatile compounds from the hay infusions making the infusions effective baits in gravid traps used for monitoring vectors of arboviral and filarial pathogens. Since Bermuda grass is abundant and widespread, it is plausible to explore infusions made from it as a potential low cost bait for outdoor monitoring of the elusive malaria vector *Anopheles gambiae s.s.*

**Methods:**

This study investigated preferential egg laying of individual *An. gambiae s.s.* in hay infusion or in tap water treated with volatiles detected in hay infusion headspace compared to tap water alone, using two-choice egg-count bioassays. Infusions were prepared by mixing 90 g of dried Bermuda grass (hay) with 24 L of unchlorinated tap water in a bucket, and leaving it for 3 days at ambient temperature and humidity. The volatiles in the headspace of the hay infusion were sampled with Tenax TA traps for 20 h and analysed using gas chromatography coupled to mass spectrometry.

**Results:**

In total, 18 volatiles were detected in the infusion headspace. Nine of the detected compounds and nonanal were selected for bioassays. Eight of the selected compounds have previously been suggested to attract/stimulate egg laying in *An. gambiaes.s.* Gravid females were significantly (p < 0.05) less likely to lay eggs in hay infusion dilutions of 25, 50 and 100 % and in tap water containing any of six compounds (3-methylbutanol, phenol, 4-methylphenol, nonanal, indole, and 3-methylindole) compared to tap water alone. The oviposition response to 10 % hay infusion or any one of the remaining four volatiles (4-hepten-1-ol, phenylmethanol, 2-phenylethanol, or 4-ethylphenol) did not differ from that in tap water.

**Conclusions:**

*Anopheles gambiae s.s.* prefers to lay eggs in tap water rather than Bermuda grass hay infusion. This avoidance of the hay infusion appears to be mediated by volatile organic compounds from the infusion. It is, therefore, unlikely that Bermuda grass hay infusion as formulated and used in gravid traps for *Culex* and *Aedes* mosquitoes will be suitable baits for monitoring gravid *An. gambiae s.s*.

## Background

Immature stages of all mosquito species (Diptera: Culicidae) are aquatic. Gravid females need to find suitable sites in or near water bodies in which to lay eggs. The aquatic stages of some mosquitoes are specialists with strong preferences for certain habitat characteristics and water qualities whilst others are found in a large variety of heterogeneous habitats [1, 2]. A number of important disease vectors, such as *Aedes aegypti, Aedes albopictus*, *Culex quinquefasciatus* and *Culex tarsalis* preferentially lay eggs in or near water bodies rich in organic matter [3–7]. For this reason, infusions that mimic these kind of oviposition sites [7] have been used as lures in gravid traps for detection and surveillance of mosquito-borne diseases such as dengue, dengue haemorrhagic fever and St. Louis encephalitis [8–14].

The sub-Saharan malaria vectors *Anopheles gambiae* sensu stricto (*s.s.*) and its sibling species *Anopheles arabiensis* are often cited to prefer small, sunlit and temporary pools for oviposition [15–18]. Nonetheless, this is an oversimplified description of their habitats [19, 20]; larvae of these species are found in a large variety of water bodies, frequently in sympatry with several species of *Culex* and *Aedes* [19, 21–24]. This might suggest that these mosquito species share some oviposition cues. Given this co-existence in habitats and the fact that hay infusions are the best known oviposition substrate for traps targeting gravid mosquitoes to date, it was imperative to investigate the response of gravid malaria vectors to hay infusions.

Hay infusions are commonly used as baits in oviposition traps since they are relatively cheap and easy to make. However, organic infusions are difficult to standardize and their efficacy for baiting mosquitoes may vary widely between batches and over time. Researchers have focused on identifying specific chemicals in the infusions that mediate the responses [25–27]. For instance, previous studies have successfully identified semiochemicals from hay infusions made from Bermuda grass that attract certain *Culex* mosquitoes [27, 28]. Millar and others [26] characterized chemicals in Bermuda grass hay infusion through solvent extraction and guided by bioassays showed that active fractions contained phenol, 4-methylphenol, 4-ethylphenol, indole, and 3-methylindole. One of the compounds, 3-methylindole, was shown to be attractive to *Cx. quinquefasciatus* in bioassays, leading to further field studies [29, 30]. In a follow-up study by Du and Millar [26], this time using electroantenography to screen volatiles, additional odorants were detected, among them nonanal. This compound has also been tested as a potential replacement for 3-methylindole in commercial baits since nonanal is less pungent and therefore more acceptable to users [31]. All these compounds have also been demonstrated to elicit responses in electroantenographic studies with gravid *An. gambiae s.s.* In addition, phenol, 4-methylphenol, 4-ethylphenol, indole, and 3-methylindole have been suggested to be attractive or simulative cues for gravid *An. gambiae s.s.* [32–36]. However, none of these compounds has been tested for actual behavioural responses in bioassays.

This study aimed to evaluate the egg-laying response of gravid *An. gambiae s.s.* to hay infusions made from Bermuda grass (*Cynodon dactylon*) and to identify the odorants released from the hay infusions that mediate the oviposition response of *An. gambiae s.s.* using behavioural two-choice cage bioassays and dynamic headspace collections analysed by gas chromatography coupled to mass spectrometry (GC–MS).

## Methods

### Preparation of hay infusions

Fresh Bermuda grass was harvested locally at the International Centre of Insect Physiology and Ecology, Thomas Odhiambo Campus (icipe-TOC), Mbita, western Kenya (0°26′06.19″ South; 34°12′53.12″ East; altitude 1149 m) and sun-dried for 48 h to make hay. Infusions were prepared by mixing 90 g of the hay with 24 L of unchlorinated tap water in a bucket. The tap water was pumped from Lake Victoria and stored in a settlement tank. The hay-water mixture was covered with a mesh (0.6 × 0.6 mm^2^), and left outdoors in a shaded area protected from rain at ambient temperature and humidity (mean daily temperature 27 ± 5 °C, relative humidity 55 ± 10 %) for 3 days. Thereafter, the infusion was filtered through a mesh (0.6 × 0.6 mm^2^). Different dilutions of the hay infusion (10, 25 and 50 %) were formulated by diluting the infusion with tap water and evaluated in behavioural assays. New batches of hay infusion were prepared for every round of bioassays.

### Dynamic headspace sampling of volatile compounds released from hay infusion

Headspace analysis was done on a single batch of undiluted infusion. This infusion was frozen at −70 °C in five 5-L aliquots in 5-L high-density polyethylene jerry cans. Each frozen aliquot was thawed at room temperature (25–28 °C) on the day of headspace sampling. The volatile compounds released from hay infusion aliquots were collected on Tenax TA traps. The traps were made from 25 mg of Tenax TA (mesh size 60/80, Supelco, Sigma-Aldrich Sweden AB, Stockholm, Sweden) packed into GERSTEL-Twister Desorption glass liners (GERSTEL, Muelheim an der Ruhr, Germany) and held in place with glass wool (Supelco, Sigma-Aldrich Sweden AB, Stockholm, Sweden). The traps were washed ten times with 2 mL of methyl-tert butyl ether (MTBE, Supelco, Sigma-Aldrich Sweden AB, Stockholm, Sweden) and then placed in a 50 °C oven with both ends covered with polytetrafluoroethylene (PTFE) tape for at least 6 h before use.

All glassware used for volatile collections was washed with a detergent, rinsed with water and acetone and then placed in a 200 °C oven overnight. Volatiles were collected from the headspace of 300 mL undiluted hay infusion with NaCl (15 g/L) added in 500 mL Erlenmeyer flasks (E-flasks). The E-flasks were fitted with gas wash bottle heads (QuickFit joined ware, Staffordshire, UK). Charcoal-filtered air was pumped through the flask at 0.5 L/min and drawn out through Tenax TA traps for 20 h. All connections were made airtight using glass and PTFE tubing and sealed with PTFE tape. After headspace collection the polymer traps were sealed with PTFE tape and stored at −70 °C. Empty E-flasks were sampled the same way and used as control for background volatiles. In total, five hay infusion samples and five empty bottle samples were collected.

### Gas chromatography-mass spectrometry (GC–MS) analyses of hay infusion headspace samples

An Agilent 7890A gas chromatograph (GC) fitted with an Agilent HP-5MS (5 % phenyl and 95 % dimethyl polysiloxane) capillary column (30 m, 250 μm internal diameter and 0.25 μm film thickness) was used for analyses. The GC was connected to an Agilent 5975C inert MSD mass spectrometer (Santa Clara, CA, USA). The GC–MS system was fitted with a GERSTEL Multi-Purpose Sampler (MPS: Gerstel GmbH & Co. KG, Mülheim an der Ruhr, Germany).

Tenax traps were thermally desorbed in splitless mode in a GERSTEL thermal desorption unit (TDU) at an initial temperature of 40 °C, then increased by 120 °C/min to 270 °C which was held for 5 min. One microlitre heptyl acetate (3.16 ng/μL) was added to the Tenax trap in the TDU unit prior to analysis. The desorbed volatiles were focused in a GERSTEL CIS inlet at 10 °C. The CIS inlet, operated in splitless mode, was then heated at a rate of 12 °C/s to 280 °C. The GC oven temperature was 40 °C at the start for a period of 1 min, then the temperature was increased by 4 °C/min to 280 °C. The final temperature was held for 3 min. Helium at a pressure of 34 psi was used as the carrier gas. The MS was set to full scan and detected mass ranges from 30 to 400 m/z with electron ionization at 70 eV and ion source temperature of 230 °C.

GC–MS data were captured and processed with the enhanced ChemStation software version E.02.01.1177 (Agilent Technologies, Santa Clara, CA, USA). All peaks that had unique retention times and/or mass spectra compared to the empty bottle control were manually integrated. Peaks present in both the empty bottle and sample collections were only integrated if they were at least twice as large in the sample. The areas of such peaks were adjusted by subtracting that of the matching peak in the empty bottle. The area of each volatile was normalized against the area of the heptyl acetate standard for each sample. All peaks with the same (adjusted) retention time and mass spectra were assigned a unique volatile identification number (vol ID). The vol ID increased with increasing retention time. Mass spectra of all vol IDs were compared to those of the National Institute of Standards and Technology 2008 library (NIST) for tentative identifications.

The identity of ten compounds (purchased from Sigma Aldrich, St. Louis, USA, >95 % pure) were confirmed using GC–MS analysis of authentic standards before they were analysed in two-choice cage bioassays. For each compound, 1 μL of a 10^−4^ M dilution in methyl tert-butyl ether [MTBE] was injected into a clean Tenax trap in the thermal desorption unit using the same GC–MS settings as described above.

### Preparation of gravid mosquitoes

All mosquitoes used for this study were supplied by the insectaries at icipe-TOC. The mosquitoes were reared following standard operating procedures [37]. Approximately 300 female and 300 male mosquitoes of the *An. gambiae s.s.* Mbita strain were selected from an adult mosquito holding cage with more than 1000 2–3 days-old mosquitoes. The selected mosquitoes were starved for about 7 h (between 12:00 and 19:00 h) before females were allowed to blood feed from a human arm for 15 min. Cotton towels saturated with tap water were placed over the cage throughout to maintain the relative humidity at 68–75 % and a temperature of 25–28 °C. Female mosquitoes that did not blood feed, as judged by the abdominal status, were removed from the cage. A vial containing 6 % glucose with a paper towel wick was introduced in the cage immediately after blood feeding for ad libitum sugar supply. A second blood meal was provided 24 h later following the same procedures. On the fifth day after the first blood meal, presumed gravid mosquitoes (based on their abdominal appearance) were selected from the cage at 16:30 by experienced technicians. These were then used for behavioural bioassays.

### Behavioural bioassays

The response of gravid *An. gambiae s.s.* to hay infusion and volatiles emitted from the infusion was evaluated using a two-choice egg-count bioassay [38]. Individual gravid females were exposed to two putative oviposition substrates in a cage. Two glass cups (Pyrex^®^, 100 mL, 70 mm diameter), one test cup, and one control cup, were set in diagonal corners of each 30 × 30 × 30 cm cage. Two types of experiments were implemented, one where two equal choices were presented in a cage and one where two different choices were presented. In the experiment with equal choices, both control and test cups were filled with 100 mL of tap water. This experiment served as the reference or baseline to which the different choice experiment was compared [38]. In the different choice experiment, the control cup was filled with 100 mL of tap water and the test cup with an equal amount of the test substrate. By systematically altering the position of the cups in each cage relative to the preceding cage, bias that could stem from the position of oviposition cups within the cages was minimized. The first test cup was randomly set in one of the four corners in the first cage. Subsequent test cups were rotated in the next possible corner in a clockwise direction relative to the position in the preceding cage. One control cup containing tap water was added in each cage diagonal to the test cup to complete the two-choice set-up. A single gravid mosquito was introduced in each cage at 18:00. The presence and number of eggs was scored for every cup the next morning at 08:00. All experiments were done in make-shift sheds at icipe-TOC at ambient conditions of temperature, humidity and light but protected from rain [37].

Table 1 provides a summary of all cage bioassays implemented including the number of mosquitoes that laid eggs (responders) over the number of mosquitoes that were tested (total number of cages set-up) and the number of rounds over which the bioassays were implemented. A round was performed over one experimental night, with a new batch of mosquitoes and a new mix of test substrate. The hay infusion was tested undiluted (100 %) and in dilutions of 50, 25 and 10 % hay infusion in tap water. The compounds were tested at various concentrations of between 0.01 and 5.00 parts per million [ppm] in tap water. A chemical was considered for further analyses if it was: (1) a dominant constituent of the Bermuda grass hay infusion headspace: 4-hepten-1-o l (97 %, Alfa Aesar, Chemtronica, Stockholm, Sweden), 4-methylphenol, 4-ethylphenol, 3-methylindole (98 %, Acros Organics, NJ, USA); (2) detected in the Bermuda grass hay infusion headspace and previously suggested to influence *Anopheles* oviposition behaviour (Table 1): 3-methyl-1-butanol, phenol, phenylmethanol, 2-phenylethanol (>99 %, Fisher Scientific, Loughborough, UK), and indole (>99 %, Acros Organics, NJ, USA); and, (3) extensively referenced in other oviposition studies with mosquitoes and identified in the headspace of Bermuda grass hay infusion in previous studies: nonanal (95 %, Sigma Aldrich, St. Louis, USA [26, 39–42]. All compounds except 4-hepten-1-ol have previously been reported to elicit electrophysiological signals from *An. gambiae s.s.* [43–51].

**Table 1:** Methodological summary of two-choice egg-count cage bioassays performed and references justifying the selection of test compounds

Test substrate	Conc of test substrate	No of rounds^a^	No of mosquitoes responding/laying eggs (total number tested)	Literature references		
				EAD signal^b^	Oviposition semio-chemical?^c^	Detected in hay infusions
Hay infusion	0^d^	8^e^	75 (75)^**e**^			
	10 %	3	126 (150)			
	25 %	3	22 (30)			
	50 %	3	25 (30)			
	100 %	3	28 (30)			
3-methyl-1-butanol	0	5	209 (250)	[44, 47, 67]	[32]	
	0.010 ppm	3	133 (151)			
	0.100 ppm	5	197 (250)			
	1.000 ppm	4	209 (300)			
4-hepten-1-ol	0	9	185 (265)	–	–	
	0.100 ppm	6	110 (148)			
	0.500 ppm	4	91 (119)			
	1.000 ppm	4	88 (116)			
	5.000 ppm	5	94 (149)			
Phenol	0	9	185 (265)	[43, 44, 67]	[33]	[25, 26]
	0.100 ppm	5	107 (146)			
	0.500 ppm	3	110 (148)			
	1.000 ppm	3	71 (90)			
	5.000 ppm	5	102 (147)			
Phenylmethanol	0	6	175 (210)		[32]	
	0.500 ppm	5	144 (175)			
	1.000 ppm	5	138 (175)			
	2.500 ppm	5	149 (210)			
	5.000 ppm	6	169 (230)			
4-methylphenol	0	11	319 (417)	[43–45, 67, 68]	[33, 35]	[25, 26]
	0.100 ppm	5	130 (208)			
	0.500 ppm	4	87 (119)			
	1.000 ppm	5	171 (238)			
	5.000 ppm	5	109 (146)			
2-phenylethanol	0	7	165 (200)		[32]	
	0.100 ppm	4	162 (200)			
	0.500 ppm	5	125 (155)			
	1.000 ppm	5	131 (155)			
	2.500 ppm	4	144 (180)			
	5.000 ppm	6	168 (195)			
Nonanal	0	4	360 (400)	[44]		[26]
	0.050 ppm	4	116 (150)			
	0.100 ppm	3	190 (250)			
	0.500 ppm	4	32 (60)			
	1.000 ppm	4	43 (60)			
4- ethylphenol	0	6	169 (220)	[44, 67]	[33]	[25, 26]
	0.100 ppm	5	110 (148)			
	0.500 ppm	3	60 (80)			
	1.000 ppm	3	60 (87)			
	5.000 ppm	3	109 (132)			
Indole	0	5	203 (430)	[43, 44, 46–49, 67, 69]	[32, 43]	[25, 26]
	0.100 ppm	4	37 (60)			
	0.500 ppm	4	40 (60)			
	1.000 ppm	5	160 (225)			
	5.000 ppm	5	158 (205)			
3-methylindole	0	8	195 (290)	[43, 44, 67]	[43]	[25, 26]
	0.010 ppm	8	102 (130)			
	0.100 ppm	8	98 (130)			
	0.500 ppm	8	110 (160)			
	1.000 ppm	8	170 (250)			

^a^A round equals a set of cages set up during the same experimental night with individual gravid female *An. gambiae s.s.* from one batch of mosquitoes and one batch of test substrates

^b^References that report electro-antennographic detection (EAD) of *An. gambiae s.s*. to this specific compound

^c^References that suggest (i.e., no bioassays performed) the specific compound to be an oviposition attractant/stimulant for *Anopheles* mosquitoes

^d^Zero (0) stands for untreated tap water, i.e., the test cups are filled with tap water (just as the control cups)

^e^The two equal-choice experiments used as reference for the hay infusion were not done in parallel to the hay infusion experiments (in contrast to all other experiments). For reference, data from 75 responders in equal-choice bioassays were randomly selected using an Excel add-in random sorter from a total of 375 responders from eight different rounds done prior to the infusion experiments

To validate the use of tap water as control substrate, 14 tap water samples (300 mL each) drawn on different dates over the study period were screened for the presence and amount of the volatile compounds examined in the bioassays. The tap water samples had been supplemented with NaCl (15 g/L) and collected as described above on Tenax traps for 20 h and thermally desorbed in the GC–MS system using the same settings as above. The resulting GC–MS data were screened for the ten test compounds using retention times and mass ions of the compounds. Of all the ten test compounds, only nonanal was detected in the tap water. Nonanal was detected in three out of the 14 samples and the concentration in these samples was lower than 0.001 ppm.

### Statistical analyses

Generalized linear models with quasibinomial distributions were used to analyse behavioural data from two-choice egg-count bioassays with hay infusions and putative semiochemicals. The proportion of eggs laid by gravid females in the test cups in the experiments with two different choices (test infusion/chemical vs control tap water) was compared to the proportion laid in test cups in the experiment with two equal tap water choices (test tap water vs control tap water). This method has been described and justified in detail previously [38]. The statistical analyses aimed at revealing if the treatment (e.g., different concentrations of grass infusion or chemicals) elicited an increase or decrease in the proportion of eggs laid in the test when given a choice of tap water as compared to the proportion of eggs laid in the test in experiments where test and control choices both contained tap water (two equal choices). The underlying assumption is that gravid females presented with equal choices respond to both in an approximately equal proportion (p = 0.5) but within a range of natural variation due to stochastic effects, which need to be accounted for by the analyses. The test treatment (tap water, infusions or chemicals) and the round number of the experiment were included in the model as fixed factors. However, no significant round differences were identified and round was removed from the final model. Mean proportions and the associated 95 % confidence intervals (CI) were predicted from the fitted models. Mosquitoes that did not lay eggs (non-responders) were excluded from the analysis since the majority that did not lay likely were not gravid [38] and, therefore, in no position to lay eggs and make a choice. On average, 75 % (73–77 %) of all females exposed in bioassays laid eggs. There was no significant difference in the proportion of non-responders between the treatments and rounds (Table 1) confirming that the lack of egg laying was not associated with the presence of the treatment. Data were analysed in R [52].

## Results

### Oviposition response of *Anopheles gambiae s.s.* to hay infusion

**Fig. 1:**
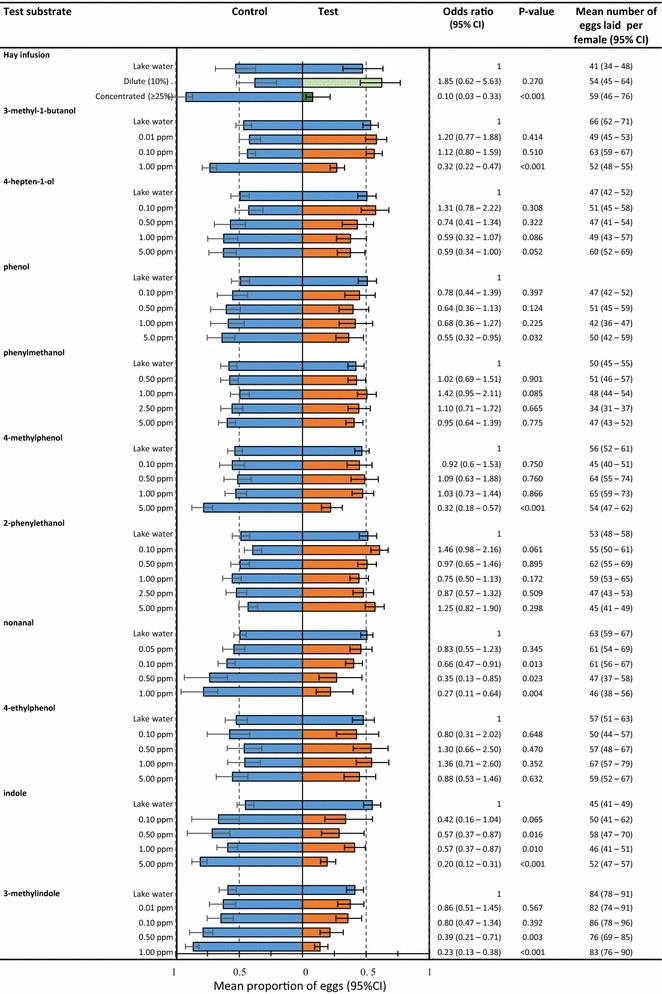
Egg-laying responses of *Anopheles gambiae s.s.* tested individually to Bermuda grass hay infusion and key organic volatiles of the infusion and the mean number of eggs laid per individual female tested. The *bar chart* shows the mean proportion of eggs laid in control and test substrates in choice egg-count bioassays, *error bars* show the 95 % confidence intervals (95 % CI). The odds ratios, including their 95 % CI and P values present the output of the statistical analysis based on generalized linear models. The experiments with tap water in both the control and test cup serve as reference based on the underlying assumption that gravid females lay an approximately equal proportion of eggs (1:1) in either test or control cup if both contain the same choice. The analysis aims to detect a statistically significant deviation from the reference distribution

When two equal tap water choices were presented, eggs were laid in similar proportions in the control and test cups (test: 0.47, 95 % CI 0.32–0.63). This result formed the baseline and validated the experimental design. The distribution of eggs in the two-choice tests with a dilute 10 % hay infusion vs tap water did not significantly differ from the baseline of tap water vs tap water (Fig. 1). In contrast, a lower proportion of eggs were laid in test cups when they contained 25 % infusions (0.11, 95 % CI 0.03–0.33), 50 % infusions (0.07, 95 % CI 0.02–0.26) and undiluted infusions (0.06, 95 % CI 0.02–0.22). There were no significant difference in the response to infusions with concentrations of 25 % and higher (p = 0.69) so the data were pooled for the final analysis. The proportion of eggs laid in the test cups with 25, 50 and 100 % hay infusion was tenfold reduced (test; OR 0.10, 95 % CI 0.03–0.33) when an alternative choice of tap water was presented compared to the proportion of eggs laid in the test cups in the experiment with both cups containing tap water (Fig. 1). The mean number of eggs laid per female per cage (irrespective of cup), was similar across experiments (Fig. 1), indicating that females did not retain eggs in the presence of any of the oviposition substrates.

### Hay infusion volatiles detected in headspace collections

**Fig. 2:**
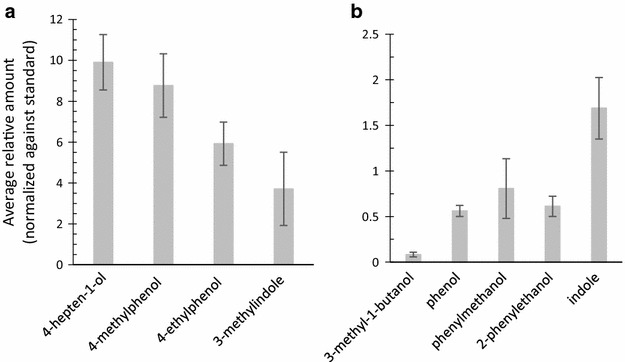
Relative amount detected in the headspace of Bermuda grass hay infusions of the volatiles evaluated in cage bioassays. Average relative amount (normalized against standard) and 95 % confidence interval. The compounds are listed in each plot in order of volatility (retention time based on GC analysis on a DB-5 column). **a** The four compounds detected in highest amount. **b** The compounds previously suggested to mediate positive oviposition responses

The four compounds detected in highest amount from the hay infusion headspace were 4-hepten-1-ol, 4-methylphenol, 3-methylindole, and 4-ethylphenol (Fig. 2) which were consequently selected for behaviour bioassays. In addition, 3-methyl-1-butanol, phenol, phenylmethanol, 2-phenylethanol, and indole were detected from the hay infusion, however in much smaller amounts (Fig. 2). The latter have all been suggested (though not tested) to mediate a positive oviposition response on *An. gambiaes.l.* in previous studies (Table 1). The identity of the compounds evaluated in bioassay was confirmed with authentic standards. Previous studies have detected nonanal in the headspace of Bermuda hay infusions. The compound was thus evaluated in addition to those detected in this study.

### Oviposition response of *Anopheles gambiae s.s.* to volatile compounds present in the hay infusion

Six out of the ten compounds tested affected the egg-laying choices of *An. gambiae s.s.* (Figure 1). The proportion of eggs laid in the test cup containing low doses of nonanal (≤0.1 ppm) was 1.5-fold decreased compared to the experiment where the test cup contained tap water. The proportion of eggs laid in indole and 3-methylindole dropped significantly in the same comparison when the test doses were raised to 0.5 ppm. This same effect was recorded for 3-methyl-1-butanol at a tenfold higher concentration (1.0 ppm) compared to the response threshold for nonanal. The chemicals phenol and 4-methylphenol were only avoided for egg-laying when presented at a concentration of 5.0 ppm, while 4-hepten-1-ol was borderline significant at the same concentration. Phenylmethanol, 2-phenylethanol or 4-ethylphenol did not affect the oviposition choices of *An. gambiae s.s* at any of the tested concentrations (between 0.01 and 5.0 ppm). The mean number of eggs laid per female did not significantly differ between experiments where the test treatment was avoided for egg laying and experiments where no preferences were exhibited (Fig. 1) confirming what was already seen for the hay infusion, that females did not retain eggs in the presence of an unsuitable oviposition substrate in the given choice scenario.

## Discussion

This study demonstrated that gravid *An. gambiae s.s.* prefer to lay eggs in tap water when the alternative choice is a Bermuda grass hay infusion, and randomly select between infusion and tap water when the infusion is highly diluted with nine parts water to one part infusion. There was only a one-in-ten chance of finding an *An. gambiae s.s.* egg in hay infusion that were little diluted (25 % and above). This is in sharp contrast to *Cx. quinquefasciatus, Culex cinereus, Culex tigripes* and *Ae. albopictus* which all prefer to lay eggs in similar infusions [3, 29]. The observed dislike for undiluted hay infusions might be the primary reason why field studies with hay infusion-baited traps rarely report trap-catches of gravid *An. gambiae s.s.* even when implemented in areas with high densities of these species [53, 54]. A notable example is the study by Mboera and others [53] in Muheza, Tanzania, where traps baited with hay infusions were evaluated when the densities of *An. gambiae s.s.* in the area were markedly high [55]. These traps, meant for *Cx. quinquefasciatus* failed to trap any *An. gambiae*.

*Anopheles gambiae s.s.* did not reject the 10 % hay infusion for oviposition. In fact the data might suggest that the mosquitoes have a slight preference for this diluted infusion. This is not surprising given that it has been shown that semiochemicals often have a concentration-dependent effect [56–58]. The fact that the diluted infusion was not avoided for egg laying indicates that a low organic matter content and the volatiles characteristic for the diluted infusion are suitable for this species. Hence, it is possible that by modifying the preparation protocol, an infusion that is more suitable as an oviposition substrate for *An. gambiae s.l.* can be obtained.

Six out of the ten tested chemicals were avoided for egg laying by gravid *An. gambiae s.s.* Few *An. gambiae s.s.* laid eggs in tap water containing indole or 3-methylindole in concentrations of 0.5 ppm and above. The indoles are well known for inducing oviposition of *Cx. quinquefasciatus* in the laboratory [25] and attracting the same species in the field [53] at a comparable concentration of 0.8 ppm. On the contrary however, Trexler and others [59] found that the compound deterred or repelled *Ae. albopictus* at a relatively high concentration of 8.3 ppm. Nonanal, a known constituent of Bermuda grass hay infusions from previous studies [26], reduced egg laying at very low concentrations in this study. This compound has previously been detected in rabbit food pellet infusions [60], which has recently been shown to be an unsuitable oviposition substrate for gravid *An. gambiae s.s.* [37]. One of the tested compounds, 3-methyl-1-butanol, has been shown to be a synergistic attractant for host-seeking *An. gambiae s.l.* and is part of novel baits used for monitoring and controlling host-seeking vectors [61]. The compound has also been suggested to be an oviposition attractant or stimulant for this species [32]. In this study however, gravid *An. gambiae s.s.* preferred to lay eggs in tap water compared to water with 3-methyl-1-butanol. Likewise, though tested in a wide range of doses, the compounds phenol, 4-methylphenol and 4-ethylphenol, that have been suggested ‘to function as oviposition attractants’ for *An. gambiae* in two studies that provided little detail of dosage [33, 35], failed to increase the egg-laying response above the response of tap water alone. Instead, phenol and 4-methylphenol led to a decrease in egg laying at the highest concentrations tested (5.0 ppm). Although the release rates of the chemicals were not quantified in this study, the results show that 4-methylphenol was present in a relatively high amount in the hay infusions tested. It is therefore possible that the natural concentration of the chemical in the hay infusion might have reached the behaviourally active levels.

It is important to highlight that suggestions from previous studies that a number of the above-mentioned compounds would induce egg laying of gravid *An. gambiae*, were based on electrophysiological results only [33, 35, 43, 62]. The results from the here-presented behavioural bioassays however show that these either mediate a reduced egg laying or no behavioural response compared to water alone. These results stress the critical importance of behavioural assays as tools for substantiating the role of compounds that elicit electrophysiological signals in insects.

The three compounds that had the lowest threshold for behaviour effects in the bioassays (nonanal, indole and 3-methylindole) are common in nature. Indole and 3-methylindole are constituents of water bodies rich in organic matter which are preferred by some species in the *Culex* and *Aedes* genera but are less likely to be chosen by *An. gambiae s.l.* if an alternative choice is available [37, 63, 64]. These compounds might therefore be important determinants for habitat separation between the species.

All the compounds identified and evaluated in this study are associated with microbial activity and metabolism. While several previous studies indicate that *An. gambiae s.s.* are sensitive to bacteria-derived odours [32, 65, 66], there is contradicting information on the role of these. Two studies suggest that microbes and their volatiles increase the egg-laying response of gravid *An. gambiae s.s.* to oviposition sites [32, 66] and another two show avoidance behaviour [37, 65]. This study adds evidence to the observation that *An. gambiae s.s.* avoids substrates rich in bacteria produced volatiles. If there are any bacteria-derived volatiles that increase oviposition responses and/or if they are dose-dependent (i.e., the 10 % hay infusion) remains elusive and warrants further investigation.

## Conclusion

Gravid *An. gambiae s.s.* females do not choose a Bermuda grass hay infusion to lay eggs when an alternative water source is available. This dislike for the hay infusion is likely mediated by volatile organic chemicals that result from bacterial metabolism within the infusion. Consequently, these infusions as formulated in this study, though used widely in gravid traps for monitoring culicine and aedine disease vectors, will not be equally useful for *An. gambiae s.s.*
